# Third-generation antipsychotics in patients with schizophrenia and non-responsivity or intolerance to clozapine regimen: What is the evidence?

**DOI:** 10.3389/fpsyt.2022.1069432

**Published:** 2022-11-29

**Authors:** Octavian Vasiliu

**Affiliations:** Department of Psychiatry, Dr. Carol Davila University Emergency Central Military Hospital, Bucharest, Romania

**Keywords:** schizophrenia, cariprazine, brexpiprazole, aripiprazole, lumateperone, treatment resistance, clozapine, third-generation antipsychotics

## Abstract

Clozapine is considered « the golden standard » for the management of treatment-resistant schizophrenia, but many patients do not present adequate responsivity even to this antipsychotic. If we add the need to strictly monitor the hematologic and cardiometabolic adverse events during each clozapine trial and the difficulty of preserving therapeutic adherence in patients with low insight, residual negative/positive symptoms, or economic challenges, then the necessity of exploring alternative interventions for these patients becomes obvious. Also, in case of intolerance to clozapine or where clozapine did not induce remission, clinicians have to find new ways to help their patients. Switching to other antipsychotics or using these agents as add-ons to clozapine are the main interventions explored in this review, for patients with schizophrenia resistant to clozapine (ultra-resistant schizophrenia, URS). When clozapine intolerance is detected, conversion to another antipsychotic with distinct pharmacologic properties or formulation (e.g., long-acting intramuscular injectable agents, LAI) may be a useful option. Third-generation antipsychotics (TGA) have been selected for their distinct pharmacodynamically profile, which allows, at a theoretical level, their use in combination with clozapine. This narrative review is based on searching four electronic databases, that retrieved 19 primary and secondary reports on aripiprazole (seven case reports or case series presenting 24 patients; nine clinical trials, and three systematic reviews/meta-analyses), two primary reports on brexpiprazole (case report and case series, *N* = 3 patients), and six primary reports on cariprazine (case reports and case series, *N* = 14 patients). Based on the information collected from these reports, which included oral and LAI formulations, the TGA most supported by evidence for the augmentation of clozapine is aripiprazole (high-and medium-quality data), followed by cariprazine (low-quality data). Brexpiprazole has not yet been systematically explored for this indication, and in the case of lumateperone, no report could be found. The efficacy of aripiprazole and cariprazine was supported in the domains of positive, negative, and general symptoms, and aripiprazole may positively impact the metabolic profile in patients with URS. Also, adding TGA may lead to a decrease in the dose of clozapine concomitantly administered. More data derived from good quality research are needed in order to confirm the circumstances of TGAs recommendation in patients with URS, either as monotherapy, or added to clozapine.

## Introduction

It is estimated that 20–30% of patients diagnosed with schizophrenia have treatment resistance, and 40–70% of these individuals have no consistent response to clozapine (1–4). There is no consensus about the definition or even the name of this clinical entity, with terms like “super-refractory schizophrenia,” “clozapine-resistant schizophrenia,” or “ultra-resistant schizophrenia” (URS) being interchangeably used in the literature (5, 6). For the purpose of this review, the term URS will be preferred because it reflects the highly resistant nature of this condition and it was fairly frequently detected in the revised manuscripts here.

The Treatment Response and Resistance in Psychosis (TRRIP) Working Group defined URS as the persistence of moderate to severe positive, negative, or cognitive manifestations in patients diagnosed with schizophrenia, after an adequate period of clozapine administration (7). Other definitions include dose range of clozapine between 200 and 500 mg/day, blood levels of clozapine ≥ 350 ng/mL, ≥ 2 months of treatment administration, with ≥ 80% of prescribed doses administered during the monitored period, moderate baseline levels of functional impairment assessed by standardized scales, moderately severe symptoms of psychosis assessed by validated instruments, less than 20% reduction of symptoms during the trial, and persistence of moderately-severe clinical manifestations and dysfunctions after the clozapine administration (5). Still, other authors include in the definition of URS more accurate limits, i.e., persistent moderately-severe BPRS total scores (≥ 45) and at least two to four positive symptoms on BPRS of ≥ 4 (moderate severity), at least a moderate score (≥ 4) on CGI scale, and persistence of GAF ≤ 40 during the last 5 years (8).

These definitions of URS are not universally accepted, therefore, the heterogeneity of inclusion criteria in trials evaluating this condition influences the quality and generalizability of their results. A meta-analysis dedicated to the subject of characteristics and definitions for URS (*n* = 71 studies, *N* = 2,731 patients) confirmed the absence of consistent use of URS definitions mentioned by guidelines in clinical practice (9). Lack of clozapine blood levels at baseline and during the trial, not mentioning the overall duration of clozapine treatment, and partial reporting on the sub-domains of the psychiatric scales administered (e.g., subscales of Positive and Negative Syndrome Scale, PANSS) were the most important aspects detected that could represent sources of bias (9). Therefore, the meta-analysis concluded that the replicability of the clinical trials’ results and their utility for clinical practice are clearly impacted negatively by this disparity between theoretically-modeled criteria for URS and the clinical reality (9).

Different case management strategies have been reported in these ultra-resistant cases, by adding antipsychotic polypharmacy, increasing the daily dose of clozapine, or combining clozapine with drugs from other pharmacological classes or with electroconvulsive therapy (ECT) (1). However, until now no strategy has been validated by randomized controlled trials as a standard intervention in patients with URS (1). More specifically, augmenting clozapine with another antipsychotic was considered to have only partial efficacy, while from the anticonvulsants class, sodium valproate and lamotrigine might lead to favorable results; the ECT could be efficient on both positive and negative dimensions, based on available data (10).

Clozapine is called “the golden standard” antipsychotic for the management of drug-resistant schizophrenia (11, 12). Although it has a low tolerability profile and requires careful monitoring of adverse events, it is widely recognized as the only efficient intervention in patients who did not respond to other antipsychotics (13). Clozapine plasma levels should also be monitored during its administration because there are consistent data suggesting this parameter correlates with the onset of therapeutic response in patients with schizophrenia (14). The usefulness of clozapine level monitoring as a predictor factor was evaluated in a systematic review (*n* = 20 studies focused on schizophrenia) (14). Clozapine levels ≥ 350 ng/ml were associated with significantly higher rates of response, but levels > 600 ng/ml did not present predictive value for therapeutic response (14). Higher mean levels of clozapine were also associated with lower rates of relapse (14). It is important to note that clozapine dose, concentration/dose ratio, or study duration was not associated with the rate of response (14). Therefore, it is important to ensure that the clozapine plasma level is preserved above 350 ng/ml before augmentation is considered (14).

Clozapine’s mechanisms of action are still considered elusive, and its uniqueness is quite challenging for researchers, as no derived compound could have been developed in more than six decades since its discovery (15). Its pharmacodynamic properties include weak D2 receptor antagonism and high affinity for D4, 5HT1A, 5HT2A, 5HT2C, 5HT6, 5HT7, α1, α2, H1, and M1-M5 receptors (2). Clozapine is metabolized mainly by CYP1A2, but also 3A4 isoforms are involved (2). The major metabolite is N-desmethylclozapine, with an activity similar to clozapine on D2 and 5HT2A receptors (2). Hypotheses for the superiority of clozapine are based on (1) mild D2 receptor occupation and/or fast dissociation; (2) increased D4 receptor affinity, and preference for D4 instead of D2; (3) high 5HT2A/D2 receptor affinity ratio (16).

Unfortunately, clozapine could hardly be considered an ideal solution for the clinical management of treatment-resistant patients, due to its low tolerability profile and considerable safety warnings (17–19). The most alarming adverse event is agranulocytosis, but many others could significantly impact these patients’ daily functionality and quality of life, i.e., sedation or somnolence, weight gain, hypersalivation, etc., (20, 21). Regarding agranulocytosis, the regulation of clozapine treatment monitoring is different in the EU and the US. A value of absolute neutrophil counts (ANC) < 1,500 mm^3^ precludes the re-challenge to clozapine in the EU, while in the US re-initiation of clozapine is allowed after the ANC increases ≥ 1,000 mm^3^ (22). Also, while no exceptions for the 1,500 mm^3^ cut-off are allowed in the EU, in the US individuals with benign ethnic neutropenia benefit from lower cut-offs (22).

A systematic review (*n* = 152 reports) exploring the tolerability of clozapine reported that most adverse events emerged within 3 months, and almost all of them were in the first 6 months post-treatment initiation (23). There were exceptions from this observation, i.e., weight gain, diabetic ketoacidosis, severe gastro-intestinal hypomotility, cardiomyopathy, seizures, and neutropenia (23). The evolution of adverse events was favorable, with spontaneous gradual reduction or after the dose decreased; rechallenge was not precluded by discontinuation due to adverse events (23). However, myocarditis, cardiomyopathy, and agranulocytosis induced by clozapine require extreme caution, and rechallenge is not advisable (23). Plasma levels of clozapine ≥ 1,000 μg/L are associated generally with an unfavorable tolerability profile (23).

Continuous clozapine treatment has been associated with a significantly lower long-term all-cause mortality when compared to other antipsychotics, according to a meta-analysis that included 24 studies reporting on 1,327 deaths of any cause in patients who received clozapine (24). Adherence to the treatment was essential for this outcome because patients who only transiently received clozapine did not present any difference in the rate of mortality when compared to other antipsychotic-treated patients (24). According to a systematic review (*n* = 28 clinical trials) that explored the effects of clozapine discontinuation, psychiatric symptoms worsened (*n* = 3 randomized trials and 14 retrospective studies) or had an uncertain evolution (*n* = 10 trials) (25). Trials focused on the effects of clozapine rechallenge reported improvements in clinical status in more than half of the patients (*n* = 4 sources), while one study reported a worse remission assessment score vs. the no-rechallenge group (25). Therefore, outcomes generally tend to be worse after clozapine discontinuation, while rechallenge may be useful (25).

Besides the clozapine use in chronic schizophrenia with treatment resistance, this antipsychotic could also be useful in patients during their early phases of psychosis. Patients with a first episode of schizophrenia spectrum disorder (FESSS) represent a specific population, which was investigated from the perspective of antipsychotic resistance. In a clinical trial (*N* = 244 with FESSS) patients received risperidone followed by olanzapine or vice versa (each of them administered for 4 weeks, and the response rate was assessed retrospectively) (26). The rate of response was 74.5% in patients who received olanzapine during the first trial, and a significantly lower response rate (less than 20%) was reported during the second antipsychotic trial (26). Therefore, a significant level of treatment resistance could be detected as early as the first episode of psychosis, prompting the need to consider the administration of clozapine in these patients, especially because the rate of remission seems to decrease with the number of antipsychotic trials.

Another three-phase study enrolled 481 patients with FESSS and involved the open-label administration of amisulpride (800 mg/day); non-responders at week 4 were included in the second phase and were randomized to amisulpride or olanzapine (≤ 20 mg/day) for another 6 weeks; non-remitters to the second treatment were given clozapine (≤ 900 mg/day) for 12 weeks in an open-label design (27). The conclusions of this trial support the use of a simple algorithm for the sequential administration of amisulpride and clozapine (27). Switching to olanzapine did not improve the outcomes, as the difference between amisulpride and olanzapine was not significant regarding the remission rate (45 vs. 44%) (27). Out of the 18 patients who reached the third phase of this trial, 28% achieved remission (27).

A comparison study that evaluated the views of consumers and their clinicians on the adverse events of clozapine concluded that patients may reconcile with this drug’s adverse effects more frequently than their treating physicians are aware of (21). Therefore, an open discussion between physicians and their patients about the tolerability of clozapine may be very useful: on one side, it could mitigate the patients’ worries about adverse events, on the other, it could avert an excessive caution that prevents many clinicians to prescribe clozapine. Third-generation antipsychotics (TGA) used in clinical practice are aripiprazole, cariprazine, brexpiprazole, and lumateperone; their common pharmacodynamic feature is partial dopamine D2 agonism. Aripiprazole has a high affinity and low intrinsic activity on D2 receptors and is also a partial serotonin 5HT1A, 5HT2A, 5HT2C, and 5HT7 agonist, with limited antagonistic properties for adrenergic α1A, histamine H1, and serotonin 5HT6 receptors; CYP3A4 and CYP2D6 are involved in the metabolism of aripiprazole and this agent has a major metabolite, dehydro-aripiprazole (28, 29). Cariprazine is a partial agonist of D2/D3 and 5HT1A receptors and an antagonist of 5HT2B and 5HT2A receptors; it is metabolized by CYP3A4 and CYP2D6 to two active metabolites (desmethyl-cariprazine and didesmethyl-cariprazine) (30). Brexpiprazole has a pharmacological profile defined by partial 5HT1A, D2, and D3 agonist activity, 5HT2A, 5HT2B, 5HT7, α1, and α2 receptors antagonism, with lower activity at histamine H1 and muscarinic M1 receptors; it is metabolized by CYP3A4 and CYP2D6, with an active metabolite, DM-3411 (28). Lumateperone is a presynaptic D2 partial agonist and post-synaptic D2 antagonist, 5HT2A receptor antagonist, and serotonin transporter inhibitor, and enhances the phosphorylation of the *N*-methyl-*D*-aspartate (NMDA) receptor- GluN2B subunits; it is metabolized by CYP3A4, 1A2, and 2C8, with more than 20 metabolites (28).

## Objectives

The main objective of this narrative review was to evaluate the data regarding the efficacy and tolerability of TGAs in the treatment of URS (defined by persistent positive, negative, cognitive, mood, and/or behavioral symptoms during a clozapine trial), either as an add-on or as an alternative to clozapine.

Another objective was to review the research exploring the efficacy and safety aspects of TGAs in patients with schizophrenia undergoing treatment with clozapine, but who could not tolerate this agent, or when a switch to another antipsychotic is required for different reasons (e.g., the patient’s non-adherence to the monitoring protocol, the expressed patient’s option for a therapeutic switch, etc.).

## Materials and methods

In order to achieve the objectives previously mentioned, four electronic databases (PubMed, Cochrane, Clarivate/Web of Science, and EMBASE) were searched using the paradigm “third generation antipsychotics” OR “aripiprazole” OR “cariprazine” OR “brexpiprazole” OR “lumateperone” AND “ultra-resistant schizophrenia” OR “clozapine-resistant schizophrenia” OR “treatment-refractory/resistant schizophrenia.” All retrieved articles were included in this review if they contained relevant data about the benefits and/or safety of TGAs in the treatment of URS, either as monotherapy or added to clozapine. No inferior time limit was established for the published papers included in the review, while the superior limit was September 2022. Also, no limitations regarding the population characteristics or type of research (primary or secondary sources) were established.

For the purpose of this narrative review, treatment-resistant cases of schizophrenia spectrum disorders (SSD) that could not tolerate efficient doses of clozapine due to significant adverse events or adherence difficulties were included in the analysis. It was considered that since these patients could not be stabilized on clozapine, the addition of a second antipsychotic (e.g., a long-acting formula) or the switch from clozapine to another agent could be possible pharmacological options. Therefore, although this may be considered a “pseudo”-ultra-resistant situation, the clinician has to make the same therapeutic choices as in the case of “true” URS (i.e., lack of efficacy of clozapine).

Also, all SSD were included in the review, since no significant differences in the pathogenesis of these disorders that could impact the choice of a certain antipsychotic versus another have been reported in the literature (31, 32).

## Results

The search retrieved six primary reports on cariprazine (case reports and case series, *N* = 14 patients), two primary reports on brexpiprazole (case report and case series, *N* = 3 patients), and 19 primary and secondary reports on aripiprazole (seven case reports or case series presenting 24 patients; nine clinical trials, and three systematic reviews/meta-analyses). All the main parameters of the reviewed papers are presented in Supplementary Table 1.

### Aripiprazole as an add-on to clozapine

A case series (*N* = 3 patients with URS) presented the clinical evolution during the aripiprazole add-on in the presence of resistant psychotic symptoms under clozapine treatment and significant adverse events of this antipsychotic (33). The patients were between 28 and 44-year-old, diagnosed with schizophrenia, and received 15 mg added to their ongoing clozapine regimen (dose range 200–300 mg/day, blood levels not assessed) (33). The favorable effects of the aripiprazole add-on strategy were visible in the following clinical aspects: less appetite and lower body weight, better glycemic control (in a patient with type 2 diabetes), more energy, higher ability to focus, lower level of negative symptoms, better overall functionality, reduction of salivation, lower level of obsessive/compulsive features, and lower level of positive symptoms (33). The only reported adverse event was transient nausea in one patient (33).

A retrospective case series (*N* = 16 patients with SSD) explored the effects of the aripiprazole and clozapine combination (34). The mean dose of clozapine was 275.56 mg/day, and these patients received aripiprazole as an add-on (mean daily dose of 11.7 mg/day) (34). The main benefit of this augmentative strategy was the improvement of metabolic status, but also reductions were observed in the general psychopathology (measured on BPRS) (34). Authors reported in these cases increases in functional autonomy, with marked reductions in social impairments (34).

A naturalistic, randomized trial enrolled 106 URS patients monitored for 12 months and evaluated the evolution of participants during treatment with aripiprazole vs. haloperidol added to clozapine (35, 60). At the endpoint, no significant difference in the rate of discontinuation at 3 months was reported between the two groups, and the change in the BPRS score was also not dependent on the treatment followed (35, 60). The only difference was the superior tolerability of aripiprazole perceived by the patients, compared to that of haloperidol, according to the Liverpool University Neuroleptic Side Effect Rating Scale scores (35, 60).

An open-label pilot trial enrolled 27 stabilized outpatients with chronic schizophrenia who presented residual manifestations during clozapine (100–900 mg/day) administration (36). The intervention consisted of oral aripiprazole (15 mg/day) and it was continued for 16 weeks (36). The symptoms improved significantly according to the PANSS scores, PANSS-Negative subscale scores, Montgomery Asberg Depression Rating Scale (MADRS), Mini-Mental State Examination (MMSE), and Quality of Life Scale (QLS) (36). The PANSS-Positive subscale did not record significant improvement during combined treatment (36). No changes were reported in the domains of extrapyramidal symptoms (assessed by the Simpson-Angus Scale and the Abnormal Involuntary Movement Scale), body weight, and prolactin levels (36).

A 24-week double-blind, randomized trial explored the effects of adding aripiprazole vs. placebo to clozapine in 31 patients diagnosed with URS (37). A beneficial effect of aripiprazole was observed on positive and general psychopathology symptoms (according to the Scale for the Assessment of Positive Symptoms, SAPS scores and BPRS scores), but the cognitive dysfunction did not change significantly (based mainly on the Wisconsin Card Sorting Test, WCST) (37). The combination was well-tolerated, with restlessness, insomnia, and nausea rarely reported (37).

A pilot study enrolled seven patients diagnosed with schizoaffective disorder or severe psychotic bipolar disorder who presented only partial response to clozapine (mean dose 292.9 ± 220.7 mg/day) (38). Aripiprazole was added (6.8 ± 3.7 mg/day) for 15 days to the current clozapine regimen in an open-label manner, and the results supported a favorable effect of this strategy: total BPRS scores decreased at a significant level, especially “thought disorder” and “anergia” significantly improved (38). No significant increase in the side effects rate was observed during the treatment period (38). The pharmacokinetics parameters were monitored (aripiprazole and clozapine levels), and they did not vary significantly throughout the trial (38).

An open-label study enrolled 11 patients with URS who received aripiprazole as an add-on to stable doses of clozapine (≥ 6 months) and were monitored for 12 weeks (39). The mean BPRS score decreased in 63% of the participants at the final study visit, and the number of adverse events did not increase compared to the baseline (39). The use of this strategy allowed for a decrease in the clozapine daily dosage (39).

In a double-blind, randomized trial that enrolled outpatients with URS (*N* = 207 participants), aripiprazole was added (5–15 mg/day) to a stable dose of clozapine in a 16-week placebo-controlled phase (40). At the end-point, aripiprazole was statistically significant to placebo for weight loss, reduction of waist circumference and BMI, but also for LDL-cholesterol values that decreased (40). No significant effects were observed on PANSS total scores between active and placebo interventions, but CGI-Improvement scores favored aripiprazole (40). The tolerability was similar between groups, but anxiety/akathisia, nausea, and headache were more frequently associated with aripiprazole (40).

A randomized, double-blind trial (*N* = 62 patients with lack of response or partial response to clozapine) explored the effects of adjuvant aripiprazole (5–30 mg/day) or placebo for 8 weeks (41). No significant difference in the primary outcome (i.e., total BPRS score at endpoint) was reported between groups, although trends toward superiority were observed for aripiprazole in the domains of negative symptoms (BPRS-negative subscale and SANS) (41). The metabolic status improved during aripiprazole intervention, with triglyceride level being lower at the endpoint; also, the prolactin level decreased in this group (41). The overall tolerability was similar between groups (41).

A Cochrane systematic review of randomized clinical trials (*n* = 3, *N* = 140 randomized participants) evaluated the efficacy of different add-on antipsychotics (risperidone, amisulpride, sulpiride, ziprasidone, and quetiapine) to clozapine and concluded, based on low-quality of evidence, that some augmentative interventions may be superior to others for specific outcomes (42). Aripiprazole may be better tolerated than first-generation antipsychotics; amisulpride and ziprasidone may lead to superior short-term improvements vs. quetiapine; risperidone may be better than sulpiride in decreasing the severity of positive symptoms and better than ziprasidone in improving mood (42). However, these differences are not enough to support the general recommendation of a certain combination strategy in URS patients (42).

According to a meta-analysis (*n* = 4 randomized controlled trials, *N* = 347 participants) exploring the efficacy and safety of aripiprazole add-on to clozapine for URS, the relative risk of discontinuation rates did not differ significantly between groups (43). The benefits of aripiprazole augmentation were only at the trend level when global psychotic, positive symptoms, and negative manifestations severity scores were analyzed (43). The metabolic profile was not significantly improved by aripiprazole augmentation, but the effects on weight change were superior vs. placebo (43). Akathisia, agitation, insomnia, and anxiety were associated significantly with aripiprazole administration (43). All trials included in the meta-analysis were of short duration, up to 24 weeks.

A meta-analysis of adjunctive atypical antipsychotics (*n* = 12 randomized controlled trials, mainly exploring risperidone and aripiprazole, but also sertindole, sulpiride, amisulpride, and ziprasidone) administered in ultra-resistant schizophrenia did not find any difference between active agents and placebo for positive symptoms (44). The results of adding atypical antipsychotics to clozapine were superior to the placebo only for negative and depressive manifestations (with a low-to-moderate effect size) (44). This meta-analysis did not explore the effects of individual antipsychotics but aggregated them into a single class, which makes difficult the interpretation of these results in a clinical context. Different agents may impact differently the core domains of schizophrenia, i.e., positive, negative, mood, aggressive, and cognitive symptoms; therefore, a distinct analysis of each antipsychotic when added to clozapine is required.

In conclusion, based on the reviewed data (two case series, seven trials, and three meta-analyses/systematic reviews) a recommendation for aripiprazole as an add-on agent to clozapine in patients with URS may be supported, with caution. The favorable effects of aripiprazole were reported on residual positive, negative, and general psychotic manifestations. The tolerability of this combination seems good (although the risk for akathisia, agitation, anxiety, and insomnia should be monitored), and the favorable effects of aripiprazole on the metabolic profile could be very useful in a population with high rates of diabetes or dyslipidemia.

### The effects of switching from clozapine to aripiprazole

This strategy was explored in patients who did tolerate the clozapine and in cases of inefficiency, low adherence, or when the patient’s preference for another antipsychotic was expressed. In a 71-year-old Caucasian male with treatment-resistant schizophrenia and comorbid alcohol use disorder, clozapine controlled most of the positive and negative symptoms during its 4-year administration (45). Treatment adherence was, however, low, and he complained of persistent discomfort due to adverse events (i.e., sialorrhea and weight gain) (45). Finally, due to a small intestine obstruction probably related to clozapine treatment, aripiprazole was initiated and titrated up to 15 mg/day while clozapine was discontinued (45). The effect of this switch on positive symptoms was favorable, and the impact on alcohol consumption was also beneficial (45). These results and an improvement in the overall functionality persisted up to 31 months after aripiprazole initiation (45).

A 37-year-old man with schizophrenia presented incomplete remission during clozapine, with persistent positive and negative symptoms, ethanol abuse, and adverse events to clozapine (i.e., somnolence) (46). Aripiprazole replaced clozapine, in a dose of 15 mg/day, for 2 months, but paranoid ideation re-appeared, therefore, it was discontinued and monotherapy with clozapine, up to 225 mg/day was re-initiated (46). Adverse effects, i.e., somnolence and sialorrhea, occurred, and a switch from clozapine to aripiprazole (10 mg/day) was again recommended, with gradual tapering of clozapine (46). Monotherapy with aripiprazole at 15 mg/day proved itself an efficient option this time and led to clinical stabilization during the 8 weeks of monitoring (46). The patient subsequently presented a re-admission due to a psychotic relapse, but in the next 3 months, he succeeds in maintaining clinical and functional stability with aripiprazole 15 mg/day as monotherapy (46).

A 42-year-old man with schizoaffective disorder presented hyperlipidemia, with total cholesterol reaching 477 mg/dl and triglyceride level 4,758 mg/dl during treatment with clozapine (47). The adherence to the clozapine regimen decreased gradually, and his condition worsened, leading to a new psychotic episode. Therefore, he was switched to aripiprazole, a strategy that led to significant improvement in the patient’s metabolic status (47). Unfortunately, the psychiatric symptoms worsened during this switch, which necessitated a switch back to clozapine (47). The lipid metabolism dysfunctions reappeared after clozapine was re-initiated (47).

In conclusion, based on very limited data (three case reports) the recommendation of switching from clozapine to aripiprazole should be very carefully analyzed in a clinical setting. Although this strategy may be beneficial, including for secondary outcomes like decreasing the craving for alcohol in patients with substance use disorders, there is always a possibility of worsening the psychotic symptoms.

### Long-acting injectable aripiprazole added to clozapine

Because poor adherence to the antipsychotic regimen, clozapine included, is a frequently reported aspect in the case management of SSD, the idea of adding LAI antipsychotics (LAIAs) to clozapine has been explored in clinical settings.

A case report presented a 21-year-old male patient diagnosed with URS (i.e., persistent residual symptoms during treatment with clozapine 300 mg/day) (48). The clozapine dose was reduced to 150 mg/day due to low tolerability (i.e., sedation, myoclonus) and the treatment regimen has been supplemented with aripiprazole LAI (up to 400 mg monthly) (48). After 12 months of monitoring, symptoms decreased by at least 50%, and no significant adverse events were reported (48).

In a 22-year-old man diagnosed with schizophrenia, clozapine 500 mg/day did not succeed in controlling persecutory delusions after the patient was already non-responsive to high doses of risperidone, olanzapine, and paliperidone palmitate in combination with clozapine (49). Aripiprazole LAI 400 mg/4 weeks was added to clozapine and the total PANSS score decreased by 25% compared to the baseline value (49). Plasma clozapine levels were preserved at 540 mg/dl (49).

A mirror-image retrospective study included adult patients diagnosed with treatment-resistant schizophrenia (*N* = 29) who received clozapine and first-generation or second-generation LAIAs (risperidone, paliperidone, aripiprazole, haloperidol, and zuclopenthixol) for ≥ 1 year after clozapine initiation (50). This study focused on the metabolic and hematologic tolerability of this pharmacological combination and concluded that adding LAIAs to clozapine is a safe strategy (i.e., the level of neutrophils, blood prolactin level, fasting blood sugar, total cholesterol, triglyceride level, HDL and LDL-cholesterol were not significantly different before and after adding LAIAs) (50). The main effectiveness outcomes (i.e., number of hospital admissions, number of relapses and days of hospitalization) were significantly improved after LAIA addition (50). The number of hospitalizations decreased by 65% vs. the previous year, the duration of hospitalization was reduced by 69%, while the number of relapses was lower by 67% (50).

Another retrospective mirror-image study included a period of analysis of 2 years pre and post-combination of clozapine with LAIAs (risperidone, paliperidone, aripiprazole, zuclopenthixol, flupenthixol, and fluphenazine) in patients with schizophrenia spectrum disorders (*N* = 20 participants) (51). The number of emergency department visits and hospital admissions post-combined therapy was significantly lower than prior to LAIAs initiation, but the reduction in hospital bed days was not significant (51). Therefore, it may be concluded that healthcare utilization could be at least partially reduced by combined clozapine and LAIAs administration (51). Also, it was reported a trend toward a decrease in the use of alcohol and illicit drugs after the combined treatment was initiated, but the sample of patients with comorbid substance use disorders was too small to have significance (*N* = 3) (51).

In conclusion, based on the available data (two case reports and two retrospective studies) adding aripiprazole LAI to clozapine may be beneficial. Besides increasing treatment adherence, this combination may help in improving the metabolic profile and decrease the psychotic symptoms and health care utilization in individuals with URS.

### Brexpiprazole as an add-on to clozapine

In a case report, brexpiprazole was added to clozapine to a hospitalized 20-year-old patient who presented with psychotic recurrence in the context of chronic schizophrenia (52). In this case, cannabis use disorder was comorbid with URS (52). The choice of the add-on agent was determined by the similarity between brexpiprazole and aripiprazole and this last agent’s properties of craving-decreasing in substance use disorders due to dopaminergic modulation of the frontal-subcortical circuits (52, 61). The patient presented a favorable evolution after 2 months of combined treatment, reflected in the Clinical Global Impression (CGI) and Brief Psychiatric Rating Scale (BPRS) score improvements (by 43 and 50%, respectively) (61). The most responsive symptoms to this add-on strategy were mood manifestations, thought disturbances, hostility, positive symptoms, negative symptoms, and craving for cannabis (61). Due to non-adherence issues, the patient was subsequently switched to clozapine combined with aripiprazole LAI (61).

The same team reported in a short communication on two cases of brexpiprazole and clozapine association in two patients with URS, both patients being subsequently switched on clozapine plus aripiprazole LAI (53). After 1 month of treatment, the clinical evolution was favorable, reflected in the PANSS scores’ improvement (over 55% total score decrease) (53). Only one patient presented at the 6 months follow-up, and his clinical status continued to improve (53).

In conclusion, existing data supporting the utility of brexpiprazole augmentation in URS patients is scarce, with only three cases being reported until now. Also, two case reports signaled the need to convert the patients on aripiprazole LAI, due to treatment adherence aspects.

### Cariprazine as an add-on to clozapine

Two case reports concluded that cariprazine might be useful as an add-on in patients with URS (54). A 29-year-old woman who previously received oral and long-acting, first, second, and third-generation antipsychotics had an inadequate response to 450 mg/day clozapine, and the trial with amisulpride 800 mg/day as an add-on failed (54). Cariprazine was chosen because although aripiprazole did not succeed in controlling the residual symptomatology in a previous trial, it was still very well-tolerated (54). Cariprazine was titrated to 3 mg/day and clinical improvement appeared after 30 days (54). The favorable evolution was maintained until the last follow-up visit, at 8 months, according to the PANSS total scores (54). Also, the body mass index (BMI) and body weight decreased, and the functionality improved, with the patient succeeding in finding a new part-time job (54). The overall tolerability was satisfactory, as no adverse effects were reported (54).

A 35-year-old man who received previously first and second-generation antipsychotics was only partially responsive to 350 mg/day clozapine (the dose could not be increased due to tolerability aspects, i.e., sedation) and presented a baseline BMI of 28.4 kg/m^2^ (54). Cariprazine was added (1.5 mg/day), and mild improvements began to appear after 3 weeks, according to the PANSS scores (mainly on positive and general sub-scales) (54). After another 5 months, the PANSS scores were lower on all subscales and the BMI value also decreased to 25.6 kg/m^2^; the doses were adjusted to 300 mg/day for clozapine and 3 mg/day for cariprazine (54).

Another case series (*N* = 5 patients with schizophrenia and treatment-resistant negative symptoms) reported on the favorable effects of clozapine augmentation using cariprazine (55). Based on the Scale for the Assessment of Negative Symptoms (SANS) scores, the favorable effects were substantiated in all cases for ≥ 6 months; the treatment adherence and clozapine blood levels, however, were not systematically assessed (55). No adverse events were reported, and in several cases, functional improvement and positive symptom amelioration were observed, although it was not objectively evaluated.

A 31-year-old male diagnosed with schizophrenia was treated with multiple antipsychotics and presented persisting psychotic symptoms, drive disorders, and negative symptoms although he was undergoing treatment with an association of clozapine and amisulpride (56). Therefore, cariprazine was added starting from 1.5 mg/day, while amisulpride was gradually tapered off (56). The final doses were 50 mg/day of clozapine and 4.5 mg of cariprazine (56). After complete remission was reached, clozapine administration was interrupted, and the patient remained on cariprazine monotherapy, with the preservation of his functionality (56).

A 41-year-old woman with URS undergoing treatment with clozapine (350 mg/day, blood concentration 533 μg/l) combined with amisulpride, flupentixol, aripiprazole, and sertraline was still presenting recurrent psychotic episodes (57). The most prominent symptoms were positive and mood manifestations (57). Cariprazine was initiated and increased up to 6 mg/day, but the patient developed dystonia corresponding to Pisa syndrome, while the positive symptoms were still significant (57). Cariprazine was discontinued at day 39, according to the patient’s expressed preference, and the increase of the clozapine dose (400 mg/day) combined with biperiden and lorazepam for Pisa syndrome was chosen (57). With this treatment, the dystonia remitted, and positive symptoms decreased; the patient was stabilized and it was possible for him to be discharged (57).

A 63-year-old man was treated with clozapine augmented with various antipsychotics (i.e., haloperidol, aripiprazole, and amisulpride) or other drugs (i.e., sertraline, valproate, and mirtazapine) during his long history of URS, but a complete remission was never achieved (57). At the index episode, especially mood symptoms, but also thinking and language disturbances and suicidal ideation were present, although the patient was receiving a combination of clozapine (850 mg/day, blood level 400 μg/l), mirtazapine, valproate, amisulpride, pipamperone, and pirenzepine (57). Cariprazine was added while amisulpride was discontinued gradually, but a Pisa syndrome occurred after 7 days of 3 mg/day (57). The cariprazine dose was reduced and gradually discontinued on day 18 (57). The patient was subsequently stabilized on a combination of clozapine, pipamperone, aripiprazole, valproate, mirtazapine, and ECT (57).

In a 45-year-old man diagnosed with treatment-resistant schizoaffective disorder and presenting a long history of treatment resistance, clozapine administered at 600 mg/day did not succeed in controlling negative symptoms and obsessive thoughts, which severely impacted his daily functioning (58). A trial with cariprazine was initiated in a slow titration regimen, up to 4.5 mg/day in combination with clozapine 450 mg/day (58). No tolerability difficulties were observed during the 9 months of monitored, combined treatment, and the functionality gradually improved (58). The clozapine dose decreased to 275 mg/day, which led to an improvement of the general status by the disappearance of several adverse events, e.g., constipation or hypersalivation (58). The CGI-S scores improved following the introduction of cariprazine in the treatment regimen (58).

In conclusion, cariprazine proved itself a useful and generally well-tolerated option when recommended as an add-on to clozapine, based on the analysis of 11 patients with URS. The favorable impact was reported mainly on negative symptoms, but also on the positive and general symptoms, and on the overall functionality. The BMI and weight gain may be positively impacted by the cariprazine add-on. Also, it is possible that adding cariprazine may help reduce the dose of clozapine administered in these patients. The main limitation of the revised papers consists of a low level of structured monitoring, using validated clinical scales. Also, a longer duration of monitoring might have been useful to consolidate the reports on cariprazine’s efficacy. The problem of monitoring adverse events is important, as severe dystonia cases were reported during cariprazine treatment (57).

### The strategy of switching from clozapine to cariprazine

The effects of switching from clozapine to cariprazine were also explored in case of intolerable adverse events or partial responsivity. A 29-year-old woman diagnosed with schizophrenia presented only partial remission and multiple adverse events–“sedation,” “cognitive blurring,” and “apathy,” after 1 year of clozapine treatment (maximum dose reached 400 mg/day) (59). The treatment adherence was confirmed, with the plasma level of clozapine ranging between 375 and 415 ng/ml (59). Cariprazine was added to clozapine and gradually increased to 6 mg/day, while the clozapine dose was reduced starting from week 2 until discontinued (59). Four months after cross-titration, the positive symptoms and mannerisms decreased significantly, together with disorganization symptoms; the overall functionality increased (59). At the 12-month-follow-up, the treatment adherence was adequate, and the psychotic symptoms were remitted, while the functionality was preserved (data confirmed by a family member) (59).

A 45-year-old male with a long history of schizophrenia (25 years) presented persistent positive, behavioral, cognitive, and negative symptoms, although his treatment adherence to clozapine (500 mg/day) was confirmed by blood concentrations (387 ng/mL) for at least 1 year (59). The medication was cross-titrated to cariprazine for 6 weeks, up to 6 mg/day, with a favorable evolution- a gradual decrease of positive symptoms, reduced psychomotor retardation, and improvement in speech content and quality for the next 6 months (59). After 1 year, negative symptoms were improved, and functionality also increased (by self-report and caregiver reports) (59).

A 25-year-old male with schizophrenia, who also had comorbid substance use disorders (cocaine, cannabis) and a low level of treatment adherence (confirmed by blood tests), was receiving treatment with clozapine 400 mg/day (59). He received long-acting injectable (LAI) paliperidone 150 mg/month in parallel with clozapine because the positive and aggressive symptoms persisted (59). After he presented a new psychotic episode, cariprazine was initiated without cross-tapering of clozapine because it was administered erratically (59). The positive symptoms decreased 5 weeks after the dose of 6 mg/day cariprazine was reached (59). The 14-month follow-up confirmed a favorable evolution of positive symptoms, therapeutic adherence was confirmed by his family, and the functionality also improved (59). The patient reported a significant decrease in the urge to use cocaine but continued to use cannabis occasionally (59).

In conclusion, switching from clozapine to cariprazine is a little supported by evidence strategy at this moment, and more data is needed before concluding its efficacy. However, the available evidence supports a favorable evolution in patients undergoing this treatment (three case reports) for up to 1 year.

## Conclusion

The review of data regarding the efficacy and tolerability of TGAs in patients with URS or clozapine intolerance included case reports, clinical trials, retrospective studies, and meta-analyses, concluding that TGAs could be useful in these cases. Unfortunately, this conclusion is based mostly on low-quality evidence (mainly case reports) for cariprazine and brexpiprazole, only aripiprazole being supported by moderate and high-quality data (randomized clinical trials, retrospective trials, meta-analyses). However, based on the retrieved clinical data, more nuanced recommendations could be formulated (see below, for each TGA). Also, it is important to analyze the risk of potential negative pharmacologic interactions in the case of antipsychotic polypharmacy, therefore, exploring these agents’ receptor binding profiles and metabolic pathways is necessary. The possibility of worsening psychosis when switching from clozapine to any other antipsychotic should also be weighed against possible benefits.

Aripiprazole may be combined with clozapine due to different pharmacodynamic profiles, which seem complementary (34). Also, there appear to be no significant interactions between aripiprazole and clozapine at the pharmacokinetic level because the first drug is metabolized by CYP2D6 and 3A4 isoforms, while clozapine by CYP1A2 (34). Aripiprazole has the advantage of multiple forms of presentation, oral and LAI, and this represents a unique advantage compared to other TGAs. Both oral and LAI formulations have been associated with favorable responses in patients with URS when added to clozapine (33–41, 60). The meta-analyses were, however, more diverse in their results referring to the efficacy of combining antipsychotics, aripiprazole included, some of them being positive (42, 44), while some of them more reserved (43). When switching from clozapine to aripiprazole was preferred, the results were mainly favorable, although reports on negative data exist (45–47). LAIAs added to clozapine were associated with favorable results in case reports and mirror-image studies, these data supporting not only clinical improvements but also significant metabolic favorable changes and a decrease in healthcare costs (48–51). One of the main advantages of combining LAIAs with clozapine refers to the increase in treatment adherence, but an additional positive effect may be seen in patients with comorbid substance use disorders (51).

Brexpiprazole could be added to clozapine because of its different receptor binding profile and lower risk of pharmacokinetic interactions. It is metabolized by CYP3A4 and 2D6, thus leaving unoccupied the CYP1A2 isoform. Based on the reviewed data, which are very limited (*n* = 3 case reports), this augmentative strategy could be efficient, but treatment adherence is challenging (52, 53, 61).

Cariprazine may decrease negative symptoms in patients with schizophrenia who are not responsive to other atypical antipsychotics (62). The effect of cariprazine on D3 receptors, auto-receptors responsible for the modulation of the phasic dopaminergic activity, and partial agonism on 5HT1A receptors may be of a certain utility in patients with incomplete response to clozapine (54). Pharmacokinetic interactions between cariprazine and clozapine are unlikely because of different primary hepatic isoenzymes involved in their metabolization (i.e., CYP3A4 and CYP2D6, and CYP1A2, respectively) (54). The good metabolic profile of cariprazine is very important when added to clozapine, a drug associated with well-known negative lipidic and glycemic metabolic effects. Adding cariprazine to clozapine may offer the possibility of reducing the dose of the second antipsychotic (54, 55). Also, in case of intolerable adverse events or lack of efficacy, switching from clozapine to cariprazine has been tried, with favorable results in case reports (*n* = 3), on positive and general symptoms of schizophrenia (59).

Lumateperone, on the other hand, may present pharmacokinetic interactions with clozapine due to its metabolization partially *via* CYP1A2 isoforms (63). However, other CYP450 isoenzymes are involved in the lumateperone metabolization (3A4, 2C8), therefore, alternate pathways are available (63, 64). The safety profile of lumateperone was evaluated in large clinical trials as good, with no clinically significant motor, endocrine, or cardiometabolic adverse events, which allows for optimism in the case of patients with schizophrenia and comorbid metabolic diseases, either primary or secondary to antipsychotic medication (64). Also, the possible use of lumateperone for major depression or bipolar depression is undergoing investigation in clinical trials, and this may bring a new perspective to the treatment of mood symptoms in schizoaffective disorder (65–67). No trial or case report regarding the use of lumateperone either as monotherapy or added to clozapine in patients with URS has been found during this search.

The main objective of this narrative review was derived from the need to answer an important practical problem: What to do when clozapine is not a solution, and the patient with schizophrenia cannot reach remission? The addition of a TGA and the switch to such an agent has been explored as possible solutions, and aripiprazole and cariprazine were found useful, in selected cases. The data for brexpiprazole and lumateperone is yet insufficient, therefore, no conclusion could be formulated in their regard.

The limitations of this paper refer to the short duration of the trials explored and to the fact the majority of the reviewed data were low-quality reports. The clozapine blood levels were not systematically assessed in all the reviewed cases. Also, the definitions of URS may vary across studies, an aspect that increases the heterogeneity of the results. Finally, this was not a systematic review, therefore, no strict inclusion and exclusion criteria were formulated and no quality of evidence was systematically assessed.

## Author contributions

The author confirms being the sole contributor of this work and has approved it for publication.
