# Supplementary material for: Third-generation antipsychotics in patients with schizophrenia and non-responsivity or intolerance to clozapine regimen: What is the evidence?
Source: Front Psychiatry. 2022 Nov 29;13:1069432. doi: 10.3389/fpsyt.2022.1069432 (PMC9744942; doi:10.3389/fpsyt.2022.1069432)
Supplement: Supplementary file 1 [file Table_1.docx]

**Table 1. The results of the reviewed primary reports regarding third-generation antipsychotics**

| **Reference** | **Type of Research** | **Population** | **Previous failed trials of antipsychotics** | **Intervention** | **Plasma CLOZ levels** | **Outcomes** | **Efficacy** | **Tolerability** | **Conclusions** |
| --- | --- | --- | --- | --- | --- | --- | --- | --- | --- |
| **Aripiprazole** | | | | | | | | | |
| [33] | Case series | 44-year-old male, URS (positive resistant symptoms + severe adverse effects to CLOZ) | Oral HAL, RIS | ARIP (15 mg/day) + CLOZ (200 mg/day), 12 weeks + one-year follow-up | Not assessed | No objective method of assessment was administered | The paranoid delusion disappeared in one month of combined treatment. Negative and mood symptoms also improved. | The sedation associated with CLOZ diminished gradually. BMI also decreased. Diabetes had a favorable evolution, and metformin was interrupted after one year of combined treatment. | The CLOZ dose could be reduced after ARI initiation, from 200 mg/day to 150 mg/day. Body weight decreased by 8 kg until week 12. After one year he lost 30 kg. |
|  |  | 30-year-old male, URS (compulsive symptoms, | Oral PIM, HAL, ARI, CLOZ (400 mg/day), 8 weeks | ARIP (15 mg/day) + CLOZ (300 mg/day), 8 weeks | Not assessed | No objective method of assessment was administered | Improvement in compulsive symptoms, but also he was more energetic and socially interactive. | Reduction of hypersalivation associated with CLOZ.  No AE related to ARI was reported. |  |
|  |  | 28-year-old male, URS (persistent psychotic symptoms) | Oral HAL, TRIFL, RIS, OLZ | ARIP (15 mg/day) + CLOZ (300 mg/day) | Not assessed | No objective method of assessment was administered | Psychotic symptoms disappeared one month after the ARI initiation. The overall functionality increased. | Somnolence decreased, and the level of activity increased. | The body weight decreased by 3.5 kg after one month. |
| [34] | Retrospective case series | N=16 patients with SSD (4 female, 12 male, mean age 45 years) and treatment resistance (clozapine included) | Not reported | ARIP (11.7 mg/day) + CLOZ (275.56 mg/day), 6 weeks | Not assessed | BPRS, blood glucose, cholesterol levels, HDL-cholesterol levels, BMI | All psychopathological and behavioral variables improved, except for PSP. Improvement of the metabolic parameters. | No AE monitoring was mentioned in the study’s protocol. | Improvement in social functioning and lipid metabolism, besides the general psychopathology scores. |
| [35] | Naturalistic, superiority RCT | N=106 patients (37 female, 69 male, mean age 40.3-41.5 years), URS (persistent positive symptoms during CLOZ treatment ≥6 months) | Not reported | ARIP+CLOZ vs. HAL+CLOZ, 12 months | Not assessed | Discontinuation rate at the endpoint was the primary outcome; BPRS score change and tolerability were secondary outcomes | No significant difference between groups in the primary outcome. No significant change in the BPRS scores at the endpoint vs. baseline was recorded. | ARIP was better tolerated than HAL, according to the LUNSERS total score. | ARIP was perceived by the patients as more tolerable than HAL. |
| [37] | OLT | N=27 outpatients (81.5% were male, mean age 41.9 years), stabilized SCHZ+ residual symptoms (positive, negative, or general) under CLOZ ≥12 months | Not reported | ARIP (15 mg/day) + CLOZ (100-900 mg/day), 16 weeks | Not assessed | PANSS, MADRS, MMSE, QLS, SAS, AIMS, PSP | Significant improvement in the mean scores for PANSS, PANSS-Negative, MADRS, MMSE, and QLS. No significant difference was detected for PANSS-Positive. | No difference in the SAS or AIMS scores was detected between groups at the endpoint. Prolactin levels and body weight were not significantly different between groups at the endpoint, either. | The results were similar for the ITT and completer analysis. |
| [38] | DBRCT | N=31 patients (17 male, 14 female, Mean age 30.7-31.9 years) with URS (persistent positive and/or negative symptoms during CLOZ 200-450 mg/day ≥1 year) | Not reported | ARIP (10 mg/day with possible increase to 15 mg/day) + CLOZ (310.7+/-73.1 mg/day) vs. placebo + CLOZ (341.2+/-77.5 mg/day), 24 weeks | Not assessed | SAPS, BPRS, SANS, SAPS, CDSS, WCST, COWAT, Stroop Test | The severity of positive and general symptoms decreased. The cognitive function did not improve significantly. | Restlessness, insomnia, and nausea were rarely reported (5, 3, and one patient, respectively). | The ARI tolerability was good, with mostly favorable effects. |
| [39] | Open-label, retrospective trial | N=7 patients (3 men, 4 women, median age 36 and 40 years, respectively) with SCHZAF or BPD who had persistent symptoms (positive, negative, or mood) during CLOZ trial | Not specified | ARIP (6.8+/-3.7 mg/day) + CLOZ (292.9+/-220.7 mg/day), 15 days | Yes, no significant variations were detected throughout the trial | BPRS, CGI, DOTES | Total BPRS scores decreased significantly; “thought disorder” and “anergia” improved significantly | No significant increase in the AE rate during the trial. | No influence of pharmacokinetic parameters over ARI positive effects could be supported, CLOZ and ARI plasma levels were controlled. |
| [40] | OLT | N=11 patients (5 female, 6 male, mean age 35.6 years) with URS (BPRS total score≥25 at baseline during CLOZ treatment) | Multiple typical and atypical antipsychotics | ARIP (26.4+/-6.4 mg/day) + CLOZ (618.8+/-160.1 mg/day), 12 weeks | The baseline CLOZ plasma level was 0.58 mg/l. This level did not vary significantly during the trial (final mean value 0.54 mg/l). | BPRS; the response to treatment was defined by a decrease of ≥20% of the total BPRS score | The response was achieved by 63.6% of the participants (7 patients). The overall BPRS score decreased significantly during the trial. | No significant increase in the AE rate during the trial. | The dose of CLOZ was significantly reduced after ARIP introduction. |
| [41] | DBRCT+ open-label extension | N=207 outpatients, URS with persistent positive, negative or general symptoms + weight gain during CLOZ treatment ≥3 months | Not reported | ARIP (5-15 mg/day) + CLOZ (mean dose 363 mg/day) vs. placebo + CLOZ (mean dose 384 mg/day), 16 weeks + 12 weeks extension (ARI + CLOZ) | Not assessed | Main outcome- mean body weight change. Secondary outcomes- PANSS, CGI, IAQ, GAF, adverse events | Both groups recorded improvements in the PANSS scores, without significant differences between them. In the extension phase, patients who switched from placebo to ARI had a higher improvement of the PANSS total score vs. those who were already on the active drug. CGI scores showed similar improvements in both groups, with a trend toward superiority at week 16 for ARI. | BMI and waist circumference decreased in the active group vs. no change in the placebo group. The benefit of ARI treatment on BMI was preserved during the open-label extension phase.  Significant improvement was detected in the LDL-cholesterol in the ARI vs. placebo group.  AEs were reported by 58.2% of the ARI-receiving patients vs. 68.8% in the placebo group. Headache, nausea, and anxiety were more frequently reported in the ARI group. | The main benefits of ARI + CLOZ treatment were on weight, BMI, and fasting cholesterol. |
| [42] | DBRCT | N=62 patients, URS with BPRS total score ≥35 or ≥2 SANS global rating item scores≥3 | Not reported | ARIP (5-30 mg/day, mean dose 15.5+/-7.1 mg/day) vs. placebo + CLOZ, 8 weeks | Not assessed | BPRS total score and subscales, SANS, CGI-S, MADRS, YBOCS, SWN | No significant difference in the primary outcome between groups. ARI treatment improved negative symptoms more than placebo in the secondary analyses (BPRS, SANS). | Prolactin and triglyceride levels were lower in the ARI group. No significant difference in the rate of AEs was detected between groups (EPS and serum glucose levels included). | The main effect of ARI was detected in the domain of negative symptoms, but the magnitude of the effect was small. |
| [43] | Systematic review (n=3 RCTs) | N=140 patients of both sexes, ≥18 years old, SSD resistant to treatment undergoing treatment with CLOZ + other antipsychotic | Not reported | CLOZ+RIS vs. CLOZ+SULP, CLOZ+RISP vs. CLOZ+ZIP, CLZO+AMI vs. CLOZ+QUE | Not included as a variable | Primary outcomes- clinical improvement. Secondary outcomes- death, discontinuation of treatment, EPS, blood dyscrasias, hypersalivation, weight gain, other AEs, hospital admission, days in the hospital, quality of life | Amisulpride and ziprasidone may lead to superior short-term clinical response vs. quetiapine; risperidone may be superior to sulpiride in reducing positive symptoms and superior to ziprasidone in improving mood. | Aripiprazole may be better tolerated than first-generation antipsychotics. | No particular combination of antipsychotics was significantly superior to others. |
| [44] | Meta-analysis (n=4 RCTs) | N=347 patients of both sexes, treatment-resistant SSD (incomplete response to CLOZ) | Not reported | ARI (11.1-15.5 mg/day) vs. placebo + CLOZ (290.6-400 mg/day), 8-24 weeks | Not included as a variable | Outcomes- the severity of psychotic symptoms- PANSS, SANS, SAPS, BPRS; cardiometabolic risk variables | The relative risk of discontinuation rates was not significantly different between groups. The benefits of ARI add-on were only at the trend level on global psychotic, positive symptoms, and negative manifestations severity scores. The metabolic profile was not significantly improved by ARI, but the effects on weight change were superior vs. placebo. | Agitation/akathisia, anxiety, and insomnia were more frequently associated with ARI vs. placebo. | The benefits of ARI add-on strategy are only at a trend level. |
| [45] | Meta-analysis (n=12 RCTs) | Patients with URS (persistent symptoms during the CLOZ trial), sample sizes between 24 and 207 | Not included as a variable | RIS, ARI, SERT, SULP, AMIS, or ZIP + CLOZ (297.1-564.7 mg/day), duration varying from 6 to 24 weeks | Not assessed | PANSS, BPRS, SAPS, SANS, CDSS, HDRS, MADRS; tolerability was assessed by discontinuation rate | The effect sizes were small/moderate on negative and mood symptoms. | No differences were reported between active and placebo-receiving groups. | There was no analysis of individual antipsychotics, but they were considered as a class. The effect of antipsychotics as add-ons was not significant only on positive symptoms. |
| [46] | Case report | 71-year-old Caucasian man, TRS + AUD + tardive dyskinesia, lack of tolerance to CLOZ | Oral PERPH, RIS; LAIAs- FLUPH, HAL | ARI (15 mg/day), 31 months | Not assessed | The clinical observation and patient reports, no validated scales were administered | Positive symptoms and alcohol consumption decreased. The overall functionality improved. Dyspnea, anxiety, and depression also resolved after the ARI switch. | Not assessed | The patient succeeded in living independently after the CLOZ to ARI switch. |
| [47] | Case report | 37-year-old Taiwanese man, URS + low tolerance to CLOZ + AUD | Oral RIS, OLZ, QUE, CLOZ, ARI | ARI (15 mg/day) monotherapy, 8 weeks- during the first trial; CLOZ monotherapy (225 mg/day), 9 months; ARI (10 mg/day) + CLOZ/ QUE, 12 weeks- during the second trial; ARI (15 mg/day) monotherapy, 28 weeks- fourth trial | Not assessed | Clinical observation, no validated scales were administered | The first trial-exacerbation of positive symptoms; the second trial- severe adverse events (somnolence, sialorrhea); the third trial- favorable clinical evolution; the fourth trial- 8 weeks of stability, followed by lack of adherence, psychotic relapse, re-hospitalization, and again 12 weeks of stability. | Not reported, except for clozapine (somnolence, sialorrhea) | The clinical evolution, in this case, is difficult to correlate with the antipsychotics administered because of low therapeutic adherence and reduced tolerability. |
| [48] | Case report | 42-year-old white man, SCHZAF with intolerance and lack of adherence to CLOZ | Not specified | Switch from CLOZ to ARI (up to 45 mg/day), 3 weeks | Not assessed | Cholesterol and triglyceride levels; clinical evaluation of psychotic symptoms (not systematically assessed by clinical scales) | The psychotic and mood symptoms worsened during the ARI administration | The lipid profile improved significantly during ARI treatment. | The switch was reversed because psychiatric symptoms worsened during ARI treatment. |
| [49] | Case report | 21-year-old man, URS + low tolerance to CLOZ | Oral HAL, RIS, ARIP; LAIAs- PAL, RIS | ARI-LAI (200-400 mg/4 weeks) + CLOZ (150 mg/day), 12 months | Not assessed | PANSS, BPRS, CGI | Psychotic symptoms decreased by 50% (PANSS) and 78% (BPRS). The CGI scores evolution reflected significant improvement. | No significant AEs were reported.  BMI remained stable. | In this case, the patient could not tolerate 300 mg/dat CLOZ due to sedation, therefore a second antipsychotic was added. |
| [50] | Case report | 22-year-old white man, URS (persistent positive symptoms during CLOZ 500 mg/day) | Oral RIS, OLZ; LAIAs- PAL + CLOZ | ARI-LAI (400 mg/4 weeks) + CLOZ (500 mg/day) | 540 mg/dl, preserved during the monitoring period | PANSS | The total score decreased by 25% vs. the baseline. | Not assessed | Significant improvement of the positive symptoms after ARI-LAI initiation. |
| [51] | Mirror-image retrospective study | N=29 patients (19 men, 10 women, mean age 30.9+/-8.47 years) with URS | Not specified | LAIAs (RIS, PAL, ARIP, HAL, ZUCL) + CLOZ, one year | Not assessed | Metabolic and hematologic parameters, number of hospitalization, number of relapses, days of hospitalization | All three efficacy outcomes were improved by the intervention. | The strategy was safe, based on neutrophil level, fasting blood sugar, total, HDL and LDL-cholesterol, triglyceride level, and prolactin blood level. | The addition of LAIAs to CLOZ was well tolerated and efficacious. The doses of LAIAs were different across this population, and no analysis focused on specific LAIAs was conducted. |
| [52] | Mirror-image retrospective study | N=20 patients (14 male, 6 female, mean age 43 years) with SSD and partial response to CLOZ | Not specified | LAIAs (RIS, PAL, ARIP, ZUCL, FLUPT, FLUPH) + CLOZ, 2 years | Not assessed | ED visits, hospital bed days, hospital admissions | ED visits and hospital admission rates decreased after the introduction of LAIAs. The symptoms scales did not record significant changes. | Not assessed | Healthcare costs may be decreased by the LAIAs + CLOZ. The severity of comorbid SUD may decrease during combined treatment. Therapeutic adherence was not assessed. |
| **Brexpiprazole** | | | | | | | | | |
| [53] | Case report | 20-year-old, URS (recurrent psychotic episodes) + SUD (cannabis) | Not reported | BREXP + CLOZ (unspecified dosing regimen), 7 weeks | Not reported | CGI, BPRS, PANSS, CDSS, MADRS, YMRS, HAMA, VASc, BARS, AIMS | After two months, BPRS scores decreased by 50% and CGI scores by 43%. Anxiety, depression, anergia, thought disturbance, hostility, positive and negative symptoms, craving -all were improved. | Akathisia was transient, while AIMS did not record any AEs. | After 7 weeks, BREX was switched to ARI-LAI, due to non-adherence. |
| [55] | Case series | A 48-year-old man, URS (“CLOZ resistance”) | Not reported | BREX+CLOZ  (unspecified dosing regimen), 1 and 6-month follow-up | Not reported | CGI, BPRS, PANSS, CDSS, VASc, BARS, BIS-11, HAMA, MADRS, YMRS, AIMS | PANSS scores decreased by 75% and 55.9%, respectively after one month.  At 6 months, only the first patient remained, and he improved further his clinical status. | No significant AE was reported. | Only one patient reached the 6-month follow-up. The other was discontinued and switched on ARI-LAI. |
|  |  | A 20-year-old man, URS(“CLOZ resistance”) + SUD (substance not specified) |  |  |  |  |  |  |  |
| **Cariprazine** | | | | | | | | | |
| [56] | Case series | 29-year-old woman, URS (persistent psychotic symptoms + body weight increase due to CLOZ) | Oral HAL, OLZ, PAL, CLOZ+AMI; LAIAs- HAL, ARI | CAR (up to 3 mg/day) + CLOZ (400 mg/day), ~32 weeks | Not assessed | PANSS | Improvement of PANSS scores was observed starting from day 30 until month 4 (last visit). The functionality also improved | No AE was reported | CAR was chosen because a previous trial with ARI was well tolerated, although not efficient. BMI and body weight decreased after ACR augmentation. |
|  |  | 35-year-old man, URS (persistent psychotic symptoms) | Oral HAL, OLZ; LAIAs- PAL | CAR (up to 3 mg/day) + CLOZ (300 mg/day), ~23 weeks | Not assessed | PANSS | Improvements in PANSS scores (positive and general subscales) were observed after 3 weeks and persisted until the last visit. The functioning also improved. | No AE was reported | The patient refused ARI because he was afraid of adverse events but accepted CAR. The BMI value also decreased after the CAR add-on. |
| [57] | Case series | 34-year-old, Eastern European woman, URS (persistent negative symptoms) | Oral ARI, AMI, QUE, OLZ, CLOZ+LUR | CAR (up to 3 mg/day) + CLOZ (275 mg/day), 12 months | Reported only initially (0.65 mg/l), prior to CAR initiation | SANS | The SANS score decreased by 56%. The residual positive symptoms were also improved (not objectively evaluated). | No AE was reported. | Body weight remained unchanged. |
|  |  | 60-year-old, white British woman, URS (persistent negative symptoms) + AUD | Oral RIS, HAL, CHLR; LAIAs- FLUPH | CAR (1.5 mg/day) + CLOZ (250 mg/day), 24 weeks | Reported only initially (0.77 mg/l), prior to CAR initiation | SANS | 41% reduction of SANS scores at the last visit | No AE was reported. | A better engagement with the medical staff was noted. |
|  |  | 23-year-old Asian British male, URS (persistent negative symptoms) | Oral ARI, OLZ, RIS;  LAIAs- ARI, PAL, HAL | CAR (1.5 mg/day) + CLOZ (325 mg/day), 12 months | Reported only initially (0.74 mg/l), prior to CAR initiation | SANS | 75% reduction of SANS scores at the last visit | No AE was reported. | A better engagement with the family members was reported. The overall functionality improved (not objectively assessed). |
|  |  | 51-year-old white British male, clozapine-resistant SCHZAF (persistent negative and suicidal ideation) | Oral AMI, QUE, OLZ | CAR (1.5 mg/day) + CLOZ (600 mg/day), 24 weeks | Reported initially (0.46 mg/l), prior to CAR initiation, and at the last visit (0.49 mg/l) | SANS | 73% reduction of SANS scores at the last visit | No AE was reported. | Body weight remained unchanged. The mood and overall functionality improved (not objectively assessed). |
|  |  | 28-year-old white British male, URS (persistent negative symptoms) + | Oral AMI, RIS, OLZ, QUE, CLOZ+AMI; LAIAs- ARI, PIPO, HAL, ZUCL | CAR (1.5 mg/day) + CLOZ (700 mg/every other day), 24 weeks | Reported initially (0.58 mg/l), prior to CAR initiation, | SANS | 56% reduction of SANS scores | No AE was reported. | A better engagement with caregivers, the patient was discharged. |
| [58] | Case report | 31-year-old male, URS (persistent negative symptoms) | Oral OLZ, CLOZ+AMI | CAR (up to 4.5 mg/day) + CLOZ (downtitrated to 50 mg/day) + AMI (300 mg/day), with gradual tapering off AMI, ≥8 weeks | Not assessed | No objective measurements were used | “Complete remission” was attained. | Not assessed | Functionality increased (not objectively assessed). CLOZ was discontinued after complete remission was achieved. |
| [59] | Case series | 41-year-old woman with URS (recurrent psychotic episodes) | CLOZ (100-375 mg/day) + AMI, FLUP, ARI (oral) | CAR (until 6 mg/day) + CLOZ (350 mg/day), 39 days | Pre-CAR initiation plasma levels of CLOZ were 533 μg/l); the last documented value was 553 μg/l. | No objective measurements were used | Initial worsening (during 3 mg/day CAR); positive symptoms persisted after CAR dose increased to 6 mg/day. | Lateral dystonia of the trunk (Pisa syndrome) | CAR was discontinued on day 39 per the patient’s preference. The Pisa syndrome was treated with biperiden and lorazepam. |
|  |  | 63-years-old man, URS (positive and negative persistent symptoms) | CLOZ (100-650 mg/day) + ARI, AMI, HAL (oral) | CAR (until 3 mg/day) + CLOZ (850 mg/day) + AMI (200 mg/day, gradually tapered off) + PIPA (140 mg/day) | The initial CLOZ level was 400 μg/l, and the final level was 527.5 μg/l | No objective measurements were used | Worsening psychotic and general symptoms | Lateral dystonia of the trunk (Pisa syndrome) | CAR was discontinued on day 18. The Pisa syndrome was treated with biperiden i.v. |
| [60] | Case report | 45-year-old man, treatment-resistant SCHZAF (severe negative symptoms and obsessive thoughts) | RIS, ZIP, CLOZ up to 650 mg/day) | CAR (up to 4.5 mg/day) + CLOZ (450 mg/day, subsequently decreased to 275 mg/day), 36 weeks | Not systematically assessed | CGI-S | The CGI-S scores improved after CAR was added to CLOZ. | No adverse events were reported | No objective measurements were used for specific psychotic dimensions. |
| [61] | Case series | 29-year-old Algerian woman, URS (persistent positive and negative symptoms) | Oral RIS, HAL, OLZ, CLOZ (400 mg/day) | Switch from CLOZ to CAR (up to 6 mg/day), gradually reduced dose of CLOZ; 16 weeks + one-year follow-up | 375-415 ng/ml (initial values) | No clinical scale was administered | Significant improvement in positive, negative, and mood symptoms and functionality (not objectively assessed) | Mild headache during the first week of the switch to CAR. | Good treatment adherence to CAR. Stable functional status at one-year follow-up. |
|  |  | 45-year-old white male, URS (poor adherence to CLOZ, frequent psychotic episodes) | Oral HAL, RIS, PAL, OLZ, ARI, CLOZ (500 mg/day) | Switch from CLOZ to CAR (up to 6 mg/day), gradually reduced dose of CLOZ; 24 weeks + one-year follow-up | 387 ng/ml (initial value) | No clinical scale was administered | Gradual improvement of positive and negative symptoms during the first six months. | CLOZ-related side effects (i.e., drowsiness, psychomotorretardation) subsided. | At one year follow-up, the functionality was preserved (by family reports). |
|  |  | 25-year-old male white male, URS (lack of adherence to CLOZ regimen, frequent psychotic episodes) + SUD (cocaine, cannabis) | Oral RIS, PAL, PAL + CLOZ (400 mg/day); LAIAs- PAL + CLOZ | CAR was initiated and increased up to 6 mg/day; no tapering of CLOZ was required due to its irregular administration; 14 months follow-up | 75 ng/ml (initial value) | No clinical scale was administered | After 2 weeks of CAR positive symptoms subsided. | The tolerability was good, and Aes related to previous treatments (PAL) gradually disappeared. | Good therapeutic adherence in long term, confirmed by family members. |

AE= adverse events; AIMS= Abnormal Involuntary Movement Scale; AMI= amisulpride; ARIP= aripiprazole; AUD= alcohol use disorder; BIS-11= Barratt Impulsivity Scale; BMI= body mass index; BARS= Barnes Akathisia Scale; BPD= bipolar disorder; BPRS= Brief Psychiatric Rating Scale; BREXP= brexpiprazole; CAR= cariprazine; CDSS= Calgary Depression Scale for Schizophrenia; CGI= Clinical Global Impression; CHLP= chlorpromazine; CLOZ= clozapine; COWAT= Controlled Oral Word Association Test; DB= double blind; DOTES= Dosage Record and Treatment Emergent Symptom Scale; ED= emergency department; EPS= extrapyramidal symptoms; FLUPH= fluphenazine; FLUPT= flupenthixol; GAF= Global Assessment of Functioning; HAL= haloperidol; HAMA= Hamilton Anxiety Rating Scale; HDRS= Hamilton Depresion Rating Scale; IAQ= Investigator Assessment Questionnaire; ITT= intent-to-treat; LAIA= long-acting injectable antipsychotics; LUNSERS= Liverpool University Neuroleptic Side Effect Rating Scale; LUR= lurasidone; MADRS= Montgomery-Asberg Depression Rating Scale; OLT= open-label trial; OLZ= olanzapine; PAL= paliperidone; PERPH= perphenazine; PIM= pimozide; PIPA= pipamperone; PIPO= pipotiazine; PSP= Personal and Social Performance Scale; QLS= Quality of Life Scale; QUE=quetiapine; RIS= risperidone; RCT= randomized controlled trial; SAS= Simpson-Angus Scale; SCHZ= schizophrenia; SCHZAF= schizoaffective disorder; SANS= Scale for the Assessment of Negative Symptoms; SAPS= Scale for the Assessment of Positive Symptoms; SERT= sertindole; SSD= schizophrenia spectrum disorders; SUD= substance use disorder; SULP= sulpiride; SWN= Subjective Well-Being Under Neuroleptics; TRFL= trifluoperazine; TRS= treatment-resistant schziophrenia; URS= ultra-resistant schizophrenia; VASc= Craving Visual Analog Scale; WCST= Wisconsin Card Sorting Test; YBOCS= Yale-Brown Obsessive Compulsive Scale; YMRS= Young Mania Rating Scale; ZIP= ziprasidone; ZUCL= zuclopenthixol
